# The inhibition of microRNA‐326 by SP1/HDAC1 contributes to proliferation and metastasis of osteosarcoma through promoting SMO expression

**DOI:** 10.1111/jcmm.15716

**Published:** 2020-08-03

**Authors:** Jiang‐Hu Huang, Yang Xu, Fei‐Yue Lin

**Affiliations:** ^1^ Shengli Clinical Medical College of Fujian Medical University Fuzhou China; ^2^ Department of Orthopaedics Fujian Provincial Hospital Fujian Medical University Fuzhou China

**Keywords:** histone deacetylase, metastasis, miR‐326, osteosarcoma, Sp1

## Abstract

Osteosarcoma (OS) is a malignant bone cancer lacking of effective treatment target when the metastasis occurred. This study investigated the implication of MicroRNA‐326 in OS proliferation and metastasis to provide the clue for the treatment of metastatic OS. This study knocked down SP1 in MG63 and 143B cells and then performed Microarray assay to find the expression of miRNAs that were influenced by SP1. MTT, EdU, wound‐healing and cell invasion assays were performed to evaluated cell proliferation and invasion. OS metastasis to lung was detected in a nude mice model. ChIP assay and DAPA were applied to determine the regulatory effect of SP1 and histone deacetylase 1 (HDAC) complex on miR‐326 expression. Human OS tissues showed lowly expressed miR‐326 but highly expressed Sp1 and HDAC. Sp1 recruited HDAC1 to miR‐326 gene promoter, which caused the histone deacetylation and subsequent transcriptional inhibition of miR‐326 gene. miR‐326 deficiency induced the stimulation of SMO/Hedgehog pathway and promoted the proliferation and invasion of 143B and MG63 cells as well as the growth and metastasis in nude mice. SP1/HDAC1 caused the transcriptional inhibition of miR‐326 gene by promoting histone deacetylation; miR‐326 deficiency conversely stimulated SMO/Hedgehog pathway that was responsible for the proliferation and metastasis of OS.

## 1 INTRODUCTION

Osteosarcoma (OS) is the most common primary bone malignancy in children and adolescents, with an incidence of 4‐5 cases per million people worldwide.^1^ Current treatment protocols in OS disease mainly include surgical resection and neoadjuvant chemotherapy. For patients with non‐metastatic OS, the 5‐year survival rate is 60%‐70%; however, there are only less than 20% 5‐year survival rates for patients with metastatic OS. The fact is that more than 20% of the patients have distant metastases at the first diagnosis.^2^, ^3^ Although advancement in OS treatment has improved the 5‐year overall survival rate, the rate of local recurrence and distant metastasis still very high.^4^, ^5^, ^6^ It is currently lack of effective treatment target when the OS metastasis occurred.^7^ Therefore, it is urgent to further elucidate the pathological mechanism underlying OS metastasis to provide clue for treatment with metastatic OS.

MicroRNAs (miRNAs) are classical small non‐coding RNAs constituted by approximately 22 nucleotides. miRNAs primarily influence the stability of mRNA by interacting with the 3′UTR.^8^ The interaction generally causes the rapid degradation of mRNA before it is translated to protein, therefore miRNAs are associated with the down‐regulation of the targeted genes.^9^ Though miRNAs play important role in the post‐transcription of genes, expression of miRNAs themselves is regulated by complex mechanisms, for example, epigenetic modifications.^10^ Epigenetic modifications which includes DNA methylation, multiple modifications of histone (eg histone acetylation and methylation) and changes of chromatin structure. Studies confirmed that epigenetic modifications play an important role in the modulation of miRNA expression.^11^, ^12^ The epigenetic modifications of miRNA during the initiation and development of cancers conversely affect various biological processes of tumour, such as the proliferation, metastasis and drug resistance.^13^, ^14^

Our preliminary study found that histone deacetylase 1 (HDAC1) and SP1 were up‐regulated in OS tissues compared to the normal tissues. HDAC1 is a key enzyme responsible for histone deacetylase.^15^, ^16^, ^17^ Although SP1 is a transcription factor, it has been found to help HDAC1‐mediated epigenetic modification. This study knocked down SP1 in two OS cell lines, followed by the miRNA Microarray detection. We found that miR‐326 was up‐regulated in both cell lines after SP1 knockdown, suggesting that miR‐326 was negatively regulated by SP1. This result was in agree with the down‐regulation of miR‐326 in OS tissues based on the data in GEO data set, given SP1 is highly expressed in OS. Interestingly, SP1, acting as a transcription factor, promotes gene expression in most cases, but SP1 negatively regulated miR‐326. We hypothesized that SP1 probably recruited HDAC1 to miR‐326 gene promoter, which caused the histone deacetylation and subsequent transcriptional inhibition of miR‐326 gene. This study performed ChIP assay to identify the hypothesis. miR‐326 was predicted to target SMO that promotes cancer proliferation and metastasis by stimulating Hedgehog signal. Therefore, down‐regulated miR‐326 is proposed to enhance the cancer‐promoting effects of SMO/Hedgehog signal. This study aimed to determine the role of a regulatory axis Sp1/HDAC1/miR‐326/SMO/Hedgehog in the proliferation and metastasis of OS.

## 2 METHODS

### 2.1 Patient samples

This study was approved by the Ethics Committee of Fujian Provincial Hospital of the Fujian Medical University in China, and every study participant gave the informed consent. A total of 20 OS tissues and their corresponding non‐cancerous tissues were obtained from the patients who underwent surgical resection of OS in the Fu Jian Provincial Hospital between 2017 and 2018. All the tissues were obtained at the time of surgery and immediately stored in liquid nitrogen until use. The clinicopathological characteristics of patients with OS were shown in Table 1.

**Table 1:** Clinicopathological characteristics of patients with OS

	Case (n)
Gender	
Male	11
Female	9
Age, y	
<21	16
≥21	4
Tumour size, cm^3^	
<2	15
≥2	5
Clinical stage	
Stage I/II	11
Stage III/IV	9
Metastasis	
Yes	3
No	17

### 2.2 Cell lines

OS cell lines (Saos‐2, U2OS, MG63 and 143B) and hFOB1.19 (SV40‐immortalized normal osteoblastic cell lines) were obtained from the Chinese Academy of Sciences Cell Bank (Shanghai, China). The OS cells were cultured in DMEM medium (Gibco) containing 10% FBS (Gibco), 100 units/mL of penicillin‐streptomycin (Invitrogen) in a humidified incubator with 5% CO_2_. hFOB 1.19 were cultured in DMEM medium, with F12, 2.5 mmol/L l‐glutamine (without phenol red), 0.3 mg/mL G418 and FBS to a final concentration of 10%.

### 2.3 RNA extraction and qRT‐PCR analyses

Total RNA was isolated from frozen tumour specimens and cell lines using the Trizol reagent (Takara). The first‐strand cDNA was generated using the miRNA Q‐PCR Detection Kit (GeneCopoeia #R0101L). U6 and GAPDH were employed as miRNA and mRNA internal control, respectively. The real‐time PCR reactions were performed in triplicate. The qRT‐PCR reaction was performed in the ABI 7500 real‐time PCR system (Applied Biosystems). The primer sequences are presented below:

**Table:** 

hsa‐miR‐326	Forward: 5′‐TTTCCTCTGGGCCCTTC‐3′;
	Reverse: 5′‐TTTGGCACTAGCACATT‐3′;
U6	Forward: 5′‐CTCGCTTCGGCAGCACA‐3′;
	Reverse: 5′‐AACGCTTCACGAATTTGCGT‐3′;
GAPDH	Forward: 5′‐TACTAGCGGTTTTACGGGCG‐3′
	Reverse: 5′‐TCGAACAGGAGGAGCAGAGAGCGA‐3′

### 2.4 Chromatin immunoprecipitation (ChIP) assay

ChIP assays were performed using Magna ChIP Assay kit (Catalog # 17‐371, Millipore) according to the manufacturer's instruction. Briefly, tissues or cells were fixed using 1% formaldehyde and harvested on ice with ChIP lysis buffer. Subsequently, the DNA was sonicated and the supernatant was collected and incubated with dynabeads protein G that was conjugated with anti‐SP1, anti‐HDAC1 and anti‐acetylated histone antibodies. IgG was used as negative control. ChIP‐derived DNA was quantified using qRT‐PCR with SYBR Green incorporation (Applied Biosystems). The primers specific for the *miR‐326* gene promoter and GAPDH are presented below:

**Table:** 

hsa‐miR‐326 promoter	Forward: 5′‐CCTGGGCTCACACAATCTTT‐3′;
	Reverse: 5′‐TCACACCTGTAATCCCAGCA‐3′;
GAPDH	Forward: 5′‐TACTAGCGGTTTTACGGGCG‐3′
	Reverse: 5′‐TCGAACAGGAGGAGCAGAGAGCGA‐3′

### 2.5 HDAC activity assay

HDAC activity was measured in nuclear lysates using HDAC1 Immunoprecipitation (IP) & Activity Assay Kit (Catalog # K342‐25; BioVision Inc, https://www.biovision.com/), following the manufacturer's protocol. Vorinostat (SAHA, MK0683, inhibitor of HDAC) was used as a control.

### 2.6 Transfection

The siRNAs specifically targeting HDAC1/2/3, SP1 and control siRNA (the sequences are depicted in Table 2) were synthesized by GenePharma (Shanghai, China) as described. Transfections were performed using the Lipofectamine 2000 kit (Invitrogen) according to the manufacturer's instructions. The double‐stranded microRNAs mimics, single‐stranded microRNAs inhibitors and their respective negative control RNAs (GenePharma) were introduced into cells at a final concentration of 2 μmol/L. The transfected cells were harvested at 24, 48 or 72 hours after transfection.

**Table 2:** The sequences of siRNAs used in this research

Gene symbol	Target Seq	Sense (5′‐3′)	Antisense (5′‐3′)
HDAC1‐SiRNA	TRCN0000004816	GCCGGUCAUGUCCAAAGUATT	UACUUUGGACAUGACCGGCTT
HDAC2‐SiRNA	TRCN0000196590	GACGGUAUCAUUCCAUAAATT	UUUAUGGAAUGAUACCGUCTT
HDAC3‐SiRNA	TRCN0000195333	CCUGCAUUACGGUCUCUAUTT	AUAGAGACCGUAAUGCAGGTT
SP1‐SiRNA	TRCN0000285151	ACCUGGAGUGAUGCCUAAUTT	AUUAGGCAUCACUCCAGGUTT
NC‐RNAi		UUCUCCGAACGUGUCACGUTT	ACGUGACACGUUCGGAGAATT

### 2.7 Microarray experiments

Microarray was performed using Human OneArray^®^ v7 Microarrays Kit (OneArray, Shanghai, China) as recommended by the manufacture. Total mRNA was extracted using a RNeasy kit (Qiagen) to prepare the Cyanine‐3 (Cy3)‐labelled cRNA. Cy3‐labelled cRNA (approximate 0.60 μg) was fragmented and hybridized to the Human Microarray for 17 hours. Slides were washed and scanned on an Agilent DNA Microarray Scanner (G2565CA) and analysed with Feature Extraction Software 10.10 (Agilent) using default parameters to obtain background‐subtracted and spatially detrended processed signal intensities.

### 2.8 Luciferase reporter assay

This study established a wild‐type pGL3 constructor (Promega) containing a putative binding site of miR‐326 on 3′UTR of SMO mRNA as well as a mutant constructor in which the putative binding site was mutant. The wild‐type and mutant constructors were transfected into MG63 cells alone or with miR‐326 mimics using Lipofectamine 2000 (Invitrogen). At 48 hours after transfection, the cells were lysed and the luciferase activity was detected by using the luciferase assay kit.

### 2.9 DNA affinity precipitation assay (DAPA)

An oligonucleotides in *miR‐326* gene promoter (wild‐type, WT: 5′‐ACTTCCTGGGCTCACACAATCTTTCCTCCTCAGCCTCCTGAGTAGCTGG‐3′) was synthesized and biotinylated at the 5′ end. In addition, the oligonucleotides with the mutation at the predicted SP‐1 binding site was also synthesized and then used as mutant type (MT: 5′‐ACTTCCTGGGCTCACACAATC**AAA**C**GT**C**AG**CAGCCTCCTGAGTAGCTGG‐3′). The WT or MT oligonucleotides (4 μg) were mixed with 20‐30 μg of cell nuclear extracts and then incubated in 400 μL of buffer D (20 mmol/L HEPES, 10% glycerol, 50 mmol/L KCl, 0.2 mmol/L EDTA, 1.5 mmol/L MgCl2, 10 μmol/L ZnCl2, 1 mmol/L DTT and 0.25% Triton X100; pH 7.9) on ice for 45 minutes. Streptavidin coated magnetosphere particles were added into the reaction buffer with further 2 hours incubation. Particles were captured using the magnetic stand and washed four times with the buffer D. The final pellet obtained was resuspended in 2 × SDS–PAGE loading buffer and boiled for 5 minutes to uncouple the oligonucleoide bound proteins. The supernatant was loaded on SDS–PAGE gel and Western analysis was performed.

### 2.10 Cell proliferation assay

Cell viability was assessed by MTT [3‐(4, 5‐dimethylthiazol‐2‐yl)‐2,5‐diphenyltetrazolium bromide] cell viability. The cells (1 × 10^3^) were seeded into 96‐well plates. The absorbance was measured using an automatic microplate reader (Tecan, NANOQUANT, Switzerland). The experiment was repeated three times.

### 2.11 5‐ethylnyl‐2′‐deoxyuridine (EdU) assay

EdU assay was performed using a Cell‐Light EdU DNA Cell Proliferation Kit (RiboBio, Shanghai, China). Cells (1 × 10^4^) were seeded in each well of 96‐well plates. After incubation with 50 mmol/L EdU for 2 hours, the cells were fixed in 4% paraformaldehyde and stained with Apollo Dye Solution. Hoechst 33342 was used to stain the nucleic acid within the cells. Images were obtained with a fluorescence microscope, and the number of EdU‐positive cells was counted.

### 2.12 Trans‐well assays

143B and MG63 cells were plated in medium without serum in the upper chamber of a trans‐well (8‐mm pore size, Millipore). The membrane of upper chamber was coated with Matrigel (BD, USA). Complete medium was subsequently added to the lower chamber. Following culture for 24 hours, the upper membrane was stained with 0.1% crystal violet in 4% Paraformaldehyde (PFA). The invading cells were quantified by counting 10 random fields under a light microscope (E200; Nikon Corporation).

### 2.13 Wound‐healing assay

Wound‐healing assay was used to further investigate cell migration. 143B and MG63 cells (5 × 10^5^) were seeded in six‐well plate and transfected with miR‐326‐mimic, SP1‐siRNA, Sp1‐siRNA + miR‐326 inhibitor or negative controls. A ‘scratch‐wound’ was created on the cell layers using a 200 μL sterile pipette tip. Cells were washed by PBS for three times and cultured in DMEM media. At time points of 0 and 48 hours, we imaged the wounds at the same position under a microscope and subsequently calculated the distance of the wound sides.

### 2.14 Western blot analysis

Cells were lysed in a lysis buffer (Sigma) containing protease inhibitor cocktails. Equal amounts of the protein samples (20 μg) were separated by 10% SDS‐PAGE and then transferred onto a nitrocellulose membrane. Proteins in the membrane were detected using following antibodies: SP1 (Abcam, ab157123), SMO (Abcam, ab38686), GLI1 (Abcam, ab49314), MMP9 (ORIGENE, AM05380PU‐N) or GAPDH (Abcam, ab125247). HRP‐linked secondary antibodies and ECL kit (Milllipore Corporation, Billerica, WBLUR0100) were further used to visualize the blots in the membrane.

### 2.15 Tumour xenograft in nude mice

The animal experiment was approved by the Ethical Committee for Animal Research of Southern Medical University (protocol number: 2011‐020). Nude mice (4‐5 weeks old, male) were purchased from the Central Animal Facility of Southern Medical University. The SP1‐siRNA was inserted in to a lentiviral plasmid, pGLVU6/GFP (GenePharma). The shRNA‐pGLVU6/GFP was transfected into 143B cells using the Lipofectamine 2000 kit (Invitrogen) according to the manufacturer's instructions. 200 mL of 143B cells (1 × 10^6^) with SP1 knockdown or not were injected into the left side on the back of each mouse. In addition, mice were injection of miR‐326 antagonist (GenePharma) through tail vein. The tumour volume was monitored on day 16. The tumour volume was calculated as *v* = *a* × *b* ^2^/2.

### 2.16 Immunohistochemistry

Formalin‐fixed and paraffin‐embedded tissues of the xenografted tumour were deparaffinized and rehydrated. Tissues were treated for 20 minutes at 100°C in an autoclave for antigen retrieval and blocked with a blocking reagent (Protein Block Serum‐Free, Dako Cytomation, Glostrup, Denmark) to avoid non‐specific reactions. Then, tissues in sections were incubated with anti‐SMO (Dilution 1:400, Abcam, ab38686) and anti‐Ki67 antibody (Dilution 1:400, ab16667, Abcam) overnight at 4°C, followed by horseradish peroxidase (HRP)‐labelled anti‐ IgG (Histofine, Simple stain MAX‐PO; Nichirei) for 30 minutes at room temperature. The sections were treated with 3, 3′‐diaminobenzidine tetrahydrochloride solution.

### 2.17 Haematoxylin and eosin (HE) staining

The lung tissues were taken from the nude mice and fixed in 10% buffered formalin. Afterwards, the tissues were embedded in paraffin, sectioned at 5 μm and stained with HE. The slides were observed and evaluated under light microscopy for histological examination.

### 2.18 Statistical analysis

Statistical analysis was performed with the Student unpaired *t* test. Statistical analysis of the data was performed using SPSS18.0 software (SPSS). A *P* value of <.05 was considered significant.

## 3 RESULTS

### 3.1 Up‐regulated SP1 and increased activities of HDACs in OS tissues and cells

**Figure 1:**
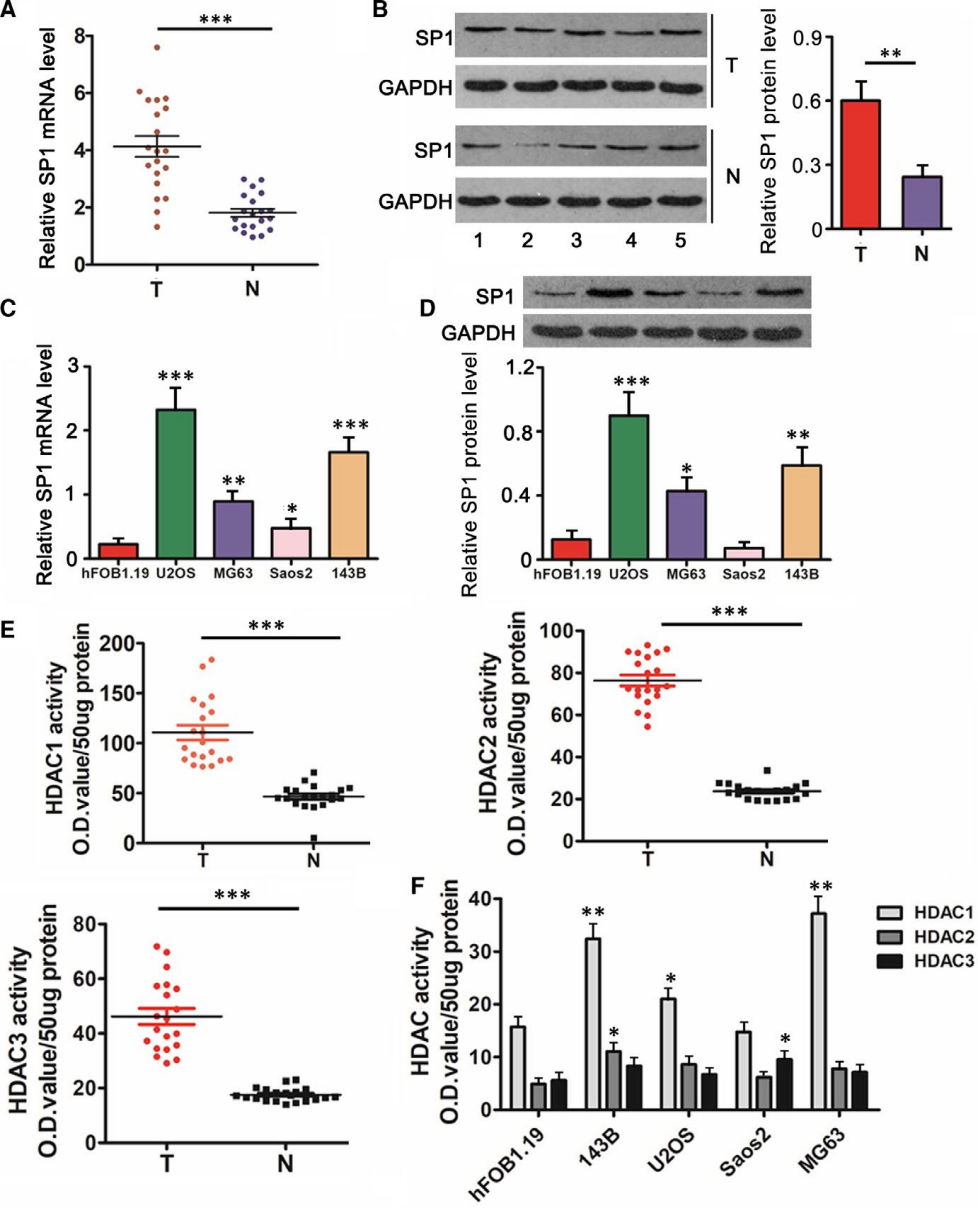
Up‐regulated SP1 and increased activities of HDACs in OS tissues and cells. SP1 expression was measured in 20 pairs of OS tissues and the matched adjacent normal tissues using RT‐qPCR (A) and western blot assays (B). Figure B shows five representive blots. SP1 expression was measured in OS cell lines and a normal osteoblastic cell line using RT‐qPCR (C) and western blot assays (D). U6 and GAPDH were employed as miRNA and mRNA internal control, respectively. miR‐326 expression was normalized to U6 expression. Activities of HDAC1/2/3 was measured in 20 pairs of OS tissues and the matched adjacent normal tissues (E) as well as in OS cell lines and a normal osteoblastic cell line (F). T means tumour tissues, N means matched adjacent normal tissues. A, B and E: **P < .01, ***P < .001 vs normal tissues. C, D and F: *P < .05, **P < .01, ***P < .001 vs hFOB1.19 cells

Results showed that both mRNA and protein levels of SP1 were up‐regulated in OS tissues compared to the corresponding normal tissues (*P* < .01 or *P* < .001, Figure 1A,B). In addition, mRNA and protein levels of SP1 were higher in the OS cell lines, such as U2OS (*P* < .001), MG63 (*P* < .05 or *P* < .01) and 143B cells (*P* < .01 or *P* < .001), than in hFOB1.19, a normal osteoblastic cell line (Figure 1C,D). Activities of all HDAC1, HDAC2 and HDAC3 were increased in OS tissues compared to the corresponding normal tissues (*P* < .001, Figure 1E). HDAC1 activity was also increased in some OS cell lines with most profound increase in MG63 (*P* < .01) and 143B (*P* < .01) cells, in comparison to that in hFOB1.19 cells (Figure 1F). The degree of the increase of HDAC2 and HDAC3 activity was less than that of HDAC1 activity, though HDAC2 and HDAC3 activities were significantly increased in 143B and Saos2 cells (*P* < .05), respectively. Based on these data, MG63 and 143B cells were used in further study.

### 3.2 The changes of miRNA expression profiles caused by SP1 knockdown

**Figure 2:**
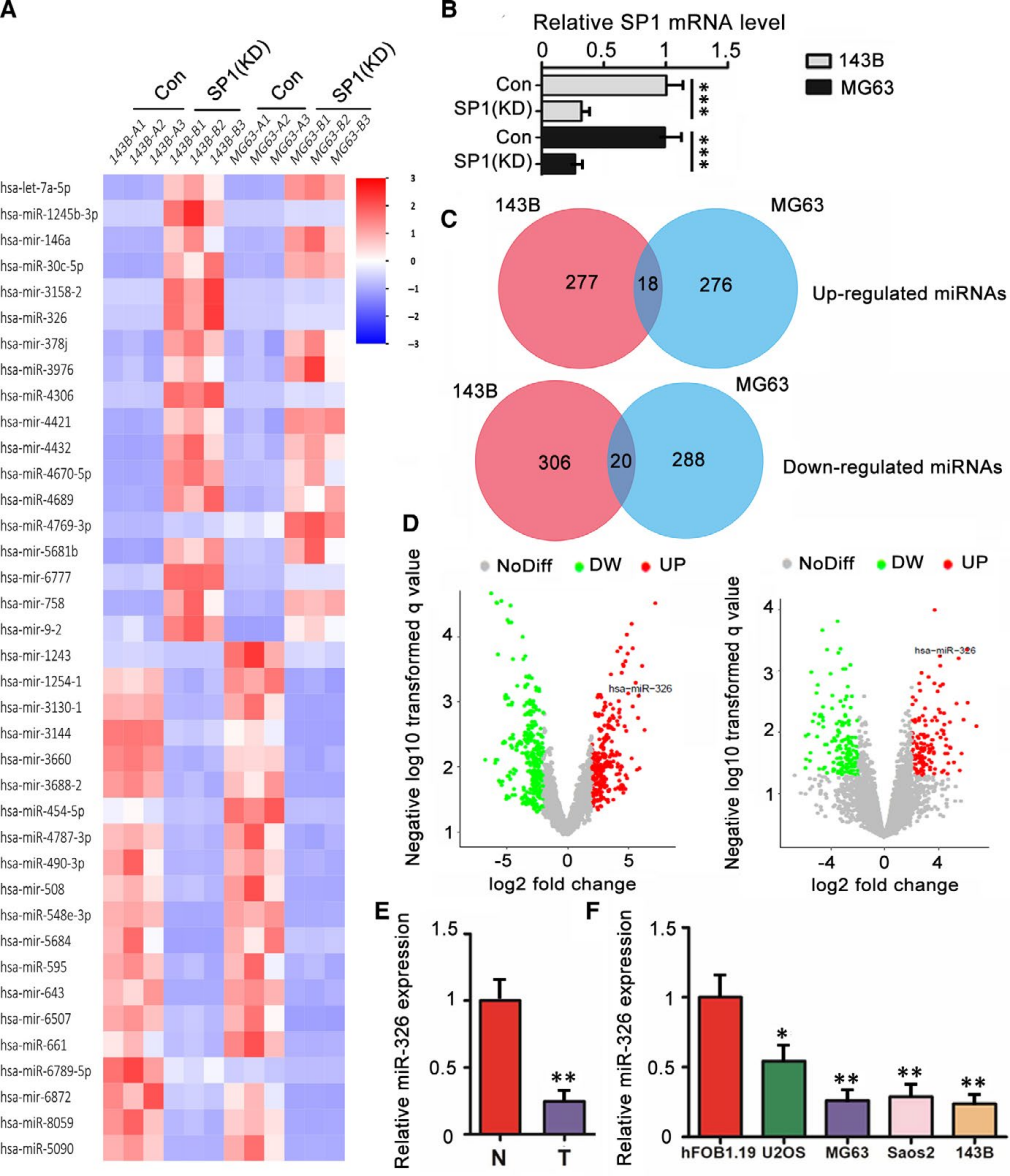
The changes of miRNA expression profiles caused by SP1 knockdown. This study knocked down SP1 in MG63 and 143B cells and then performed Microarray assay to find the expression of miRNAs that were influenced by SP1. A, Heatmap of Microarray assay. B, SP1 expression level in MG63 and 143B cells was assessed by PCR assay after transfecting SP1‐siRNA. C, Venn diagram shows the 18 up‐regulated miRNAs and 20 down‐regulated miRNAs in both MG63 and 143B cells after SP1 knockdown. D, Volcano plots show the significantly changed miRNAs in MG63 and 143B cells after SP1 knockdown. The expression of miR‐326 in osteosarcoma tissues and the matched adjacent normal tissues (E) as well as in OS cell lines and a normal osteoblastic cell line (F). U6 and GAPDH were employed as miRNA and mRNA internal control, respectively. KD means knockdown; T means tumour tissues; N means matched adjacent normal tissues. *P < .05, **P < .01 vs hFOB1.19 cells

This study knocked down SP1 in MG63 and 143B cells and then performed Microarray assay to find the expression of miRNAs that were influenced by SP1 (Figure 2A,B). The data showed that expression of 18 miRNAs was increased in both MG63 and 143B cells after SP1 knockdown (Figure 2C). There were 20 miRNAs whose expression were down‐regulated in both MG63 and 143B cells after SP1 knockdown. Among the up‐regulated miRNAs (Figure 2D), miR‐326 was predicted to target SMO during bioinformatics analysis (http://www.targetscan.org/vert_72/). Since SMO‐mediated Hedgehog signalling plays critical roles in tumour occurrence and progression, this study aimed to investigate the regulatory effect of SP1/miR‐326 axis on Hedgehog signalling. miR‐326 expression was down‐regulated in OS tissues compared to the corresponding normal tissues (*P* < .01, Figure 2E). Moreover, in comparison to hFOB1.19, all Saos‐2, U2OS, MG63 and 143B cells showed decreased expression of miRNA‐326 (*P* < .05 or *P* < .01, Figure 2F).

### 3.3 miRNA‐326 expression was regulated by SP1 and HDAC1

**Figure 3:**
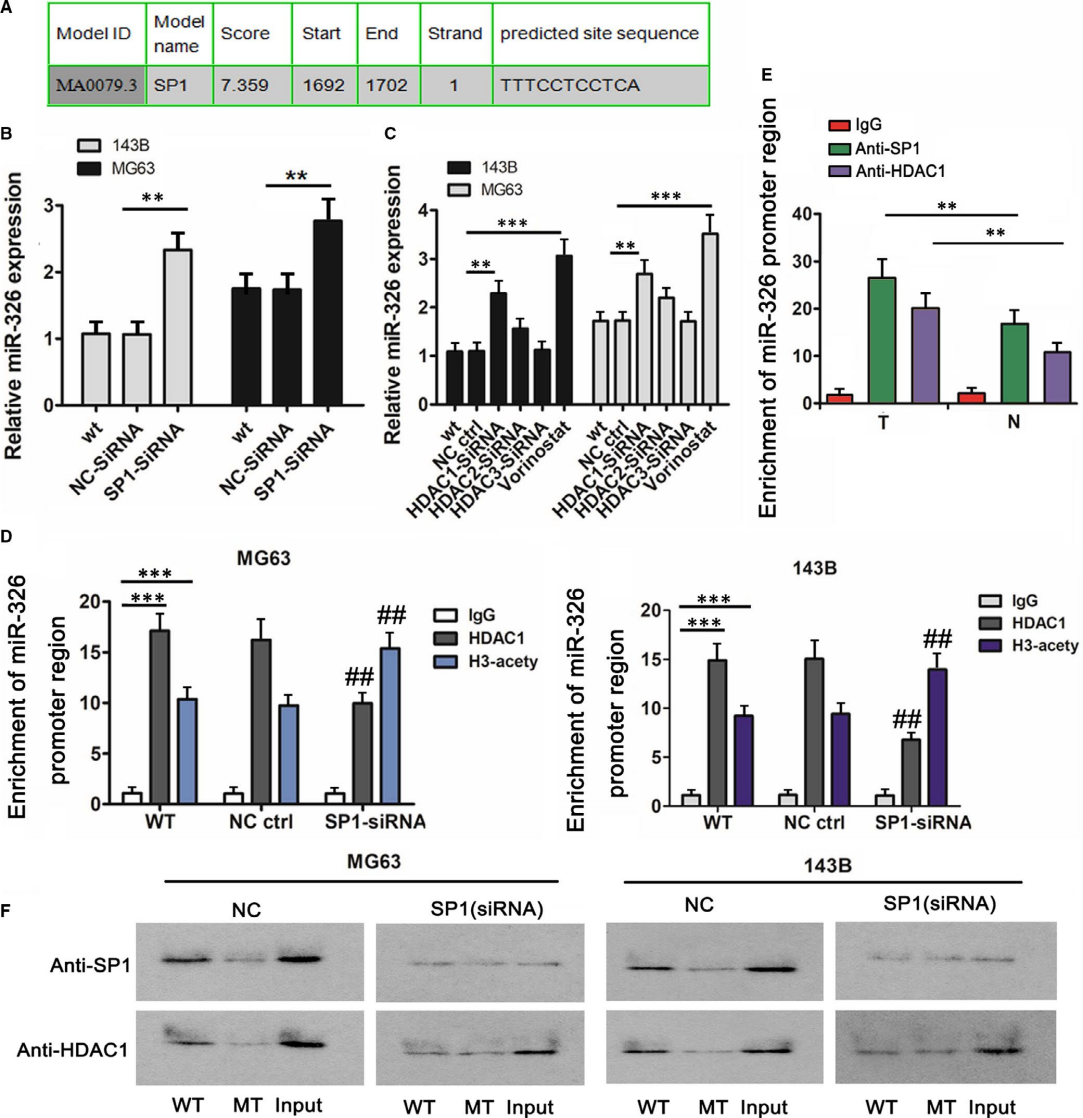
miRNA‐326 expression was regulated by SP1 and HDAC1. A, Using an open online Jaspar database (http://jaspar.genereg.net), bioinformatics analysis showed that SP1 was able to bind to the promoter of miRNA‐326 gene. B, miRNA‐326 expression was increased in MG63 and 143B cells 72 h after SP1 knockdown. C, miRNA‐326 expression was increased in MG63 and 143B cells 72 h after HDAC1 knockdown or treatment with HDAC inhibitor. D, ChIP assay was performed to determine the interaction between SP1, HDAC1 and acetylated histone 3 proteins and the promoter of *miRNA‐326* gene. Both antibodies of HDAC1 and acetylated histone 3 proteins extracted the DNA sequence of *miRNA‐326* gene promoter. Knockdown of SP1 decreased the DNA enrichment in HDAC1 protein complex, but increased the DNA enrichment in acetylated histone 3 protein complex. E, ChIP assay was also performed to detect the enrichment of *miRNA‐326* gene promoter in SP1 and HDAC1 proteins in OS tissues and the matched adjacent normal tissues. F, In DAPA, WT and MT of *miRNA‐326* promoter oligonucleotides were designed to pull down SP1 and HDAC1 proteins in MG63 and 143B cells. SP1 and HDAC1 proteins were pulled down by the WT oligonucleotides, but almost not by MT oligonucleotides. SP1 knockdown notably decreased the abundance of HDAC1 protein pulled down by the WT oligonucleotides. B and C: **P < .01, ***P < .001 vs WT group. D: ***P < .001 vs IgG group; ^##^ *P* < .01 vs WT group that did not undergo transfection. E: **P < .01 vs adjacent normal tissues. All above studies were repeated three times

Using an open online Jaspar database (http://jaspar.genereg.net), bioinformatics analysis showed that SP1 was able to bind to the promoter of miRNA‐326 gene (Figure 3A). As indicated by PCR, SP1 knockdown conversely caused the up‐regulation of miRNA‐326 in MG63 and 143B cells (*P* < .01, Figure 3B). Similarly, HDAC1 knockdown also increased miRNA‐326 expression in MG63 and 143B cells (*P* < .01, Figure 3C). However, the increase caused by depletion of HDAC2 and HDAC3 was not significant. Treatment with HDAC inhibitor, vorinostat, induced the up‐regulation of miRNA‐326, as well (*P* < .001). We performed ChIP assay to determine the interaction between SP1, HDAC1 and acetylated histone 3 proteins and the promoter of miRNA‐326 gene. Both antibodies of HDAC1 and acetylated histone 3 proteins extracted the DNA sequence of miRNA‐326 gene promoter (*P* < .001, Figure 3D). Knockdown of SP1 decreased the DNA enrichment in HDAC1 protein complex (*P* < .01), but increased the DNA enrichment in acetylated histone 3 protein complex (*P* < .01). Furthermore, using ChIP assay, we found that the enrichment of *miRNA‐326* gene promoter is higher in OS tissues compared to the corresponding normal tissues (*P* < .01, Figure 3E). We also conducted DAPA to further determine the interaction between SP1 and HDAC1 proteins and miRNA‐326 promoter oligonucleotides. SP1 and HDAC1 proteins were pulled down by the WT oligonucleotides, but almost not by MT oligonucleotides (Figure 3F). SP1 knockdown notably decreased the abundance of HDAC1 protein pulled down by the WT oligonucleotides.

### 3.4 SP1/miR‐326 modulated proliferation, migration and invasion of OS cells

**Figure 4:**
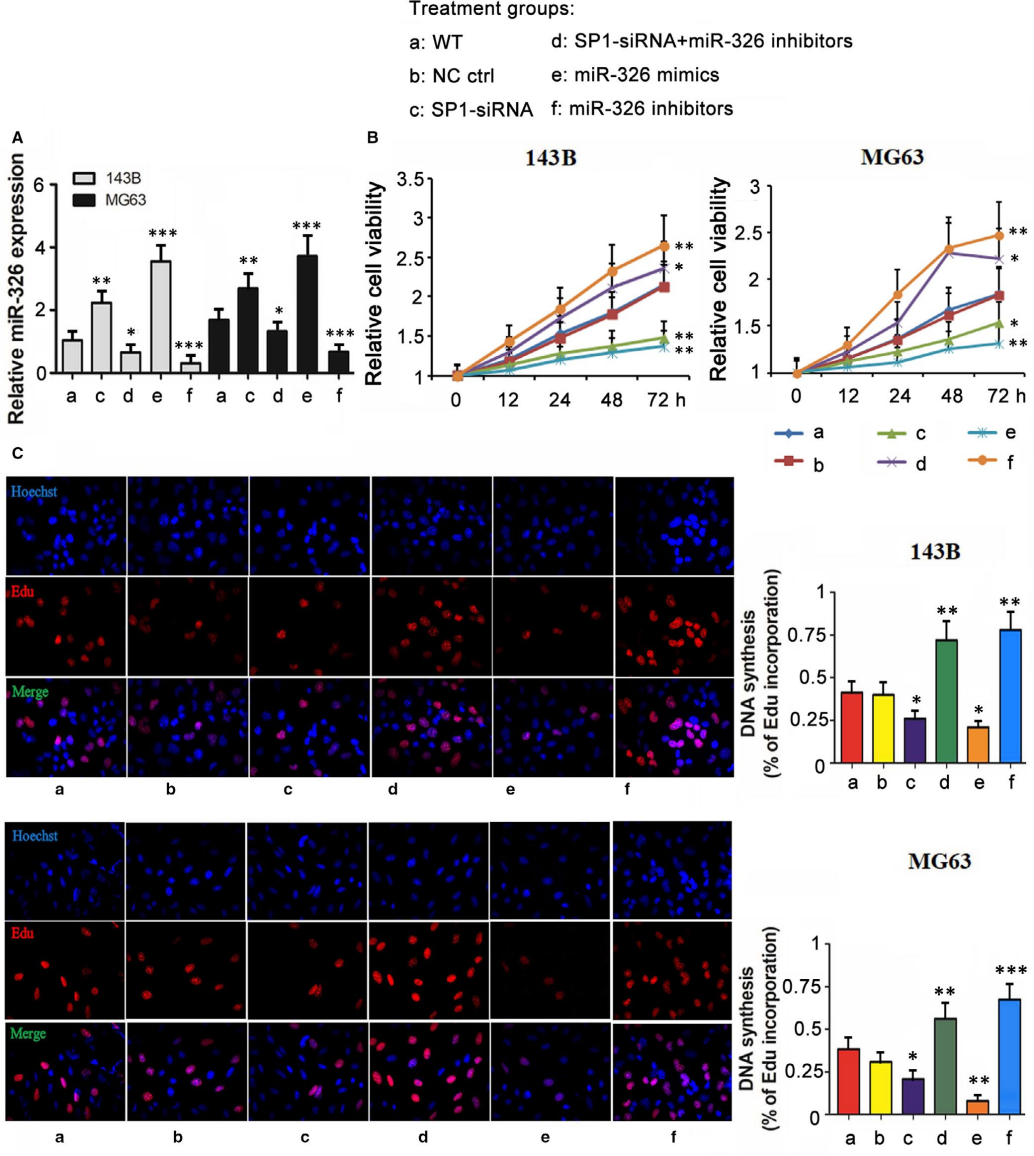
SP1/miR‐326 modulated viability and proliferation of OS cells. MG63 and 143B cells were transfected with siRNA‐SP1, siRNA‐SP1 together with miR‐326 inhibitors, miR‐326 mimics, miR‐326 inhibitors and negative control. A, PCR was performed to measure miR‐326 expression level. Cell viability and proliferation were evaluated using MTT (B) and Edu assays (C), respectively. Statistical analysis was performed at the time point of 72 h in MTT assay and at 48 h in Edu assay. *P < .05, **P < .01 vs WT group that did not undergo transfection

To determine the regulatory effects of SP1/miR‐326 axis on OS proliferation and invasion, we transfected SP1‐siRNA, SP1‐siRNA + miR‐326 inhibitors, miR‐326 inhibitors and miR‐326 mimics into MG63 and 143B cells. Although SP1‐siRNA promoted miR‐326 expression (*P* < .01, Figure 4A), co‐transfection with miR‐326 inhibitors consequently down‐regulated miR‐326 expression (*P* < .05). Individual transfection with miR‐326 inhibitors and mimics decreased and increased miR‐326 expression, respectively (*P* < .001). SiRNA‐mediated knockdown of SP1 or the mimics‐mediated increase of miR‐326 suppressed MG63 and 143B cell viability at the time points of 72 hours (*P* < .01, Figure 4B). However, the suppressive effect on MG63 and 143B cell viability resulted from SP1 depletion was reversed by inhibition of miR‐326 expression by the inhibitor (*P* < .05). Treatment with miR‐326 inhibitors alone promoted the cell viability as well (*P* < .01). Edu assay was performed to further determine the effects of SP1 and miR‐326 on OS cell proliferation. Both SP1 deficiency and miR‐326 overexpression attenuated the proliferation of MG63 and 143B cells (*P* < .05 or *P* < .01, Figure 4C), but SP1 deficiency with miR‐326 knockdown conversely enhanced cell proliferation (*P* < .01). Treatment with miR‐326 inhibitors alone promoted the cell proliferation as well (*P* < .01 or *P* < .001).

**Figure 5:**
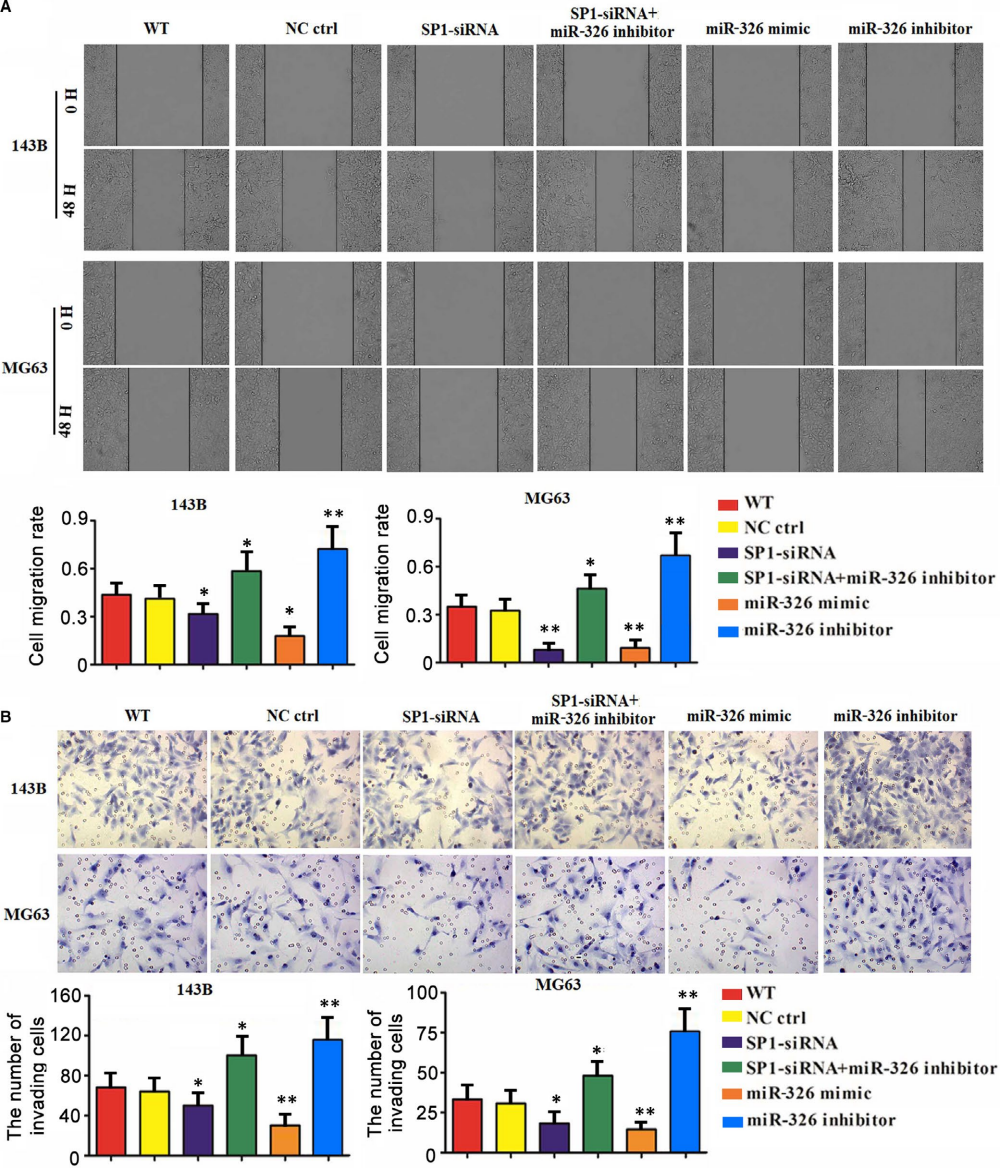
SP1/miR‐326 modulated migration and invasion of OS cells. MG63 and 143B cells were transfected with SiRNA‐SP1, SiRNA‐SP1 together with miR‐326 inhibitors, miR‐326 mimics, miR‐326 inhibitors and negative control. Cell migration (A) and invasion (B) were evaluated using scratch and transwell assays, respectively. *P < .05, **P < .01 vs WT group that did not undergo transfection

The invasion and migration of MG63 and 143B cells were suppressed after SP1 knockdown and miR‐326 overexpression (*P* < .05 or *P* < .01, Figure 5A,B). Knockdown of SP1 and miR‐326 at the same time strengthened the invasion and migration of MG63 and 143B cells (*P* < .05). miR‐326 knockdown alone also strengthened the invasion and migration of MG63 and 143B cells (*P* < .01).

### 3.5 SP1/miR‐326 modulated SMO/GLI1/MMP‐9 signalling pathway

**Figure 6:**
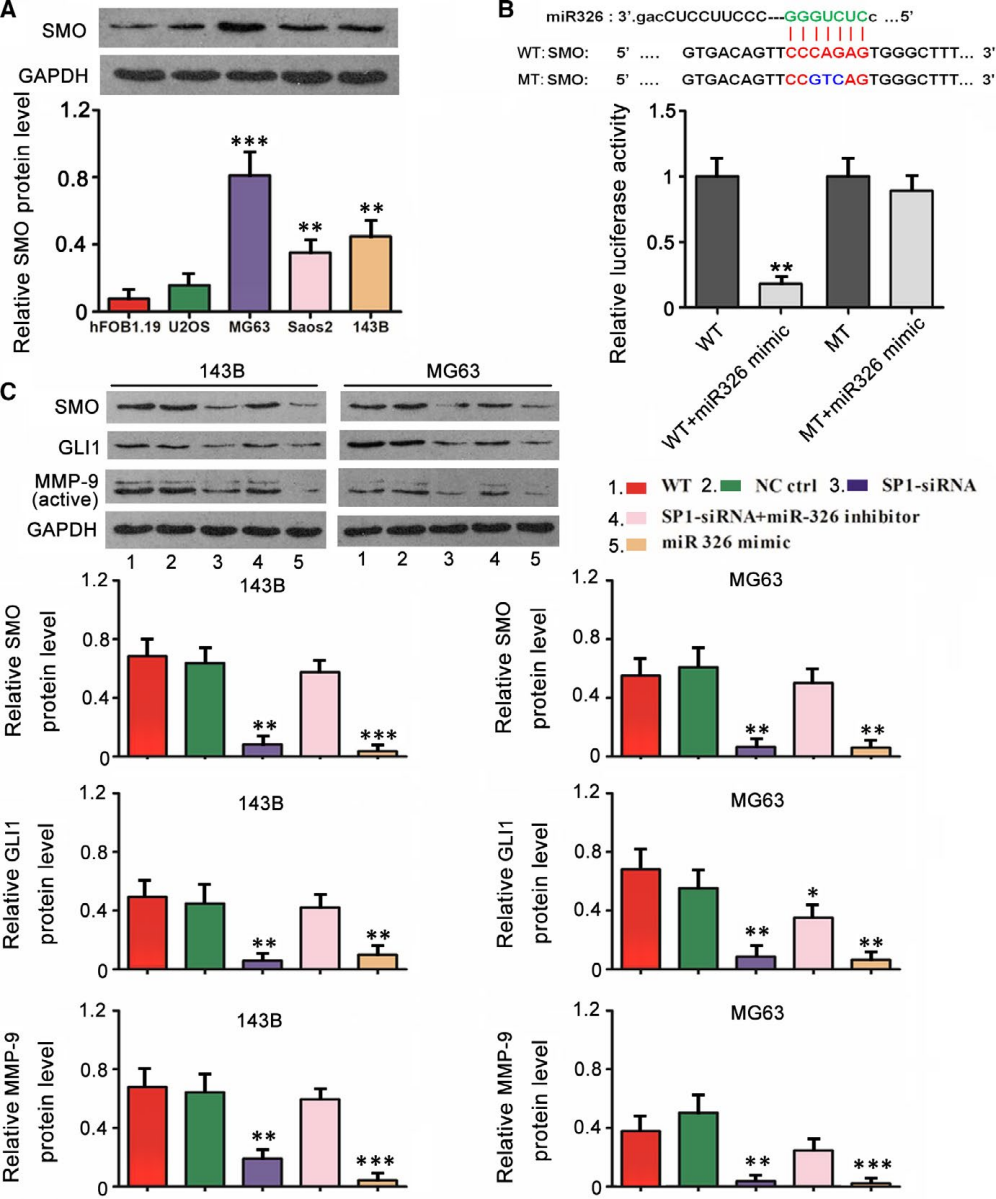
SP1/miR‐326 modulated SMO/GLI1/MMP‐9 signalling pathway. A, Western blot was conducted to measure SMO expression in OS cell lines and a normal osteoblastic cell line. B, Dual luciferase reporter assay was conducted to explore whether SMO targeted miR‐326 directly. Transfection of miR‐326 mimics inhibited the WT luciferase activity, while had not effect on MT luciferase activity. C, MG63 and 143B cells were transfected with siRNA‐SP1, siRNA‐SP1 together with miR‐326 inhibitor, miR‐326 mimics and negative control. Western blot was conducted to measure SMO, GLI1 and MMP‐9 protein level. A, *P < .05, **P < .01, ***P < .001 vs hFOB1.19 cells. B and C, *P < .05, **P < .01, ***P < .001 vs WT group that did not undergo transfection

Using miRanda and Targetscan, bioinformatics analysis showed that oncogene SMO is a potential target of miR‐326. We initially performed western blot to measure SMO expression in OS cell lines. In comparison to hFOB1.19, U2OS, MG63 and 143B cells showed increased protein level of SMO (*P* < .01 or *P* < .001, Figure 6A). Dual luciferase reporter assay was conducted to explore whether SMO targeted miR‐326 directly. Transfection of miR‐326 mimics inhibited the WT luciferase activity (*P* < .01, Figure 6B), while had not effect on MT luciferase activity. The regulatory effect of SP1/miR‐326 axis on SMO/GLI1/MMP‐9 signalling pathway was evaluated using western blot assay. SP1 knockdown and miR‐326 overexpression reduced protein levels of SMO, GLI1 and MMP‐9 (*P* < .01 or *P* < .001, Figure 6C). The reduction of SMO, GLI1 and MMP‐9 protein levels resulted from SP1 knockdown was abolished by miR‐326 knockdown.

### 3.6 SP1/miR‐326 modulated the proliferation and metastasis of 143B cells in nude mice

**Figure 7:**
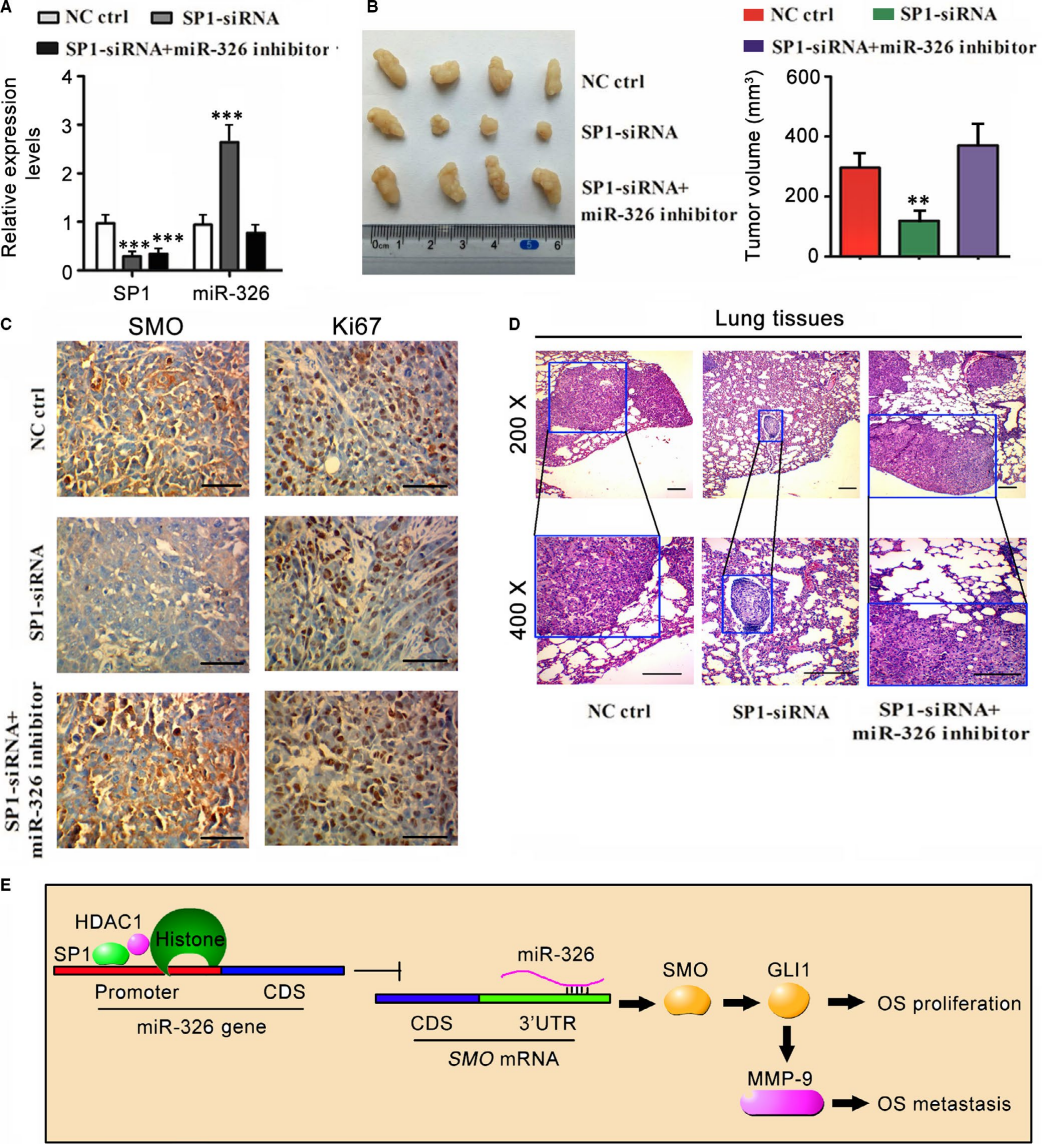
SP1/miR‐326 modulated the proliferation and metastasis of 143B cells in nude mice. A, Transfection with SP1‐siRNA vector decreased SP1 but increased miR‐326 expression in 143B‐cell‐xenografted tumour, as indicated by the results form PCR assay. Treatment with miR‐326 inhibitor (antagonist) abolished the increased of miR‐326 expression caused by SP1 knockdown. B, SP1 knockdown inhibited 143B tumour growth in nude mice. However, the inhibition of tumour growth was abolished by the reduction of miR‐326. C, As indicated by immunohistochemistry, SMO and Ki67 protein abundances were decreased with SP1 knockdown. D, In lung tissues, SP1 knockdown was associated to smaller size of 143B tumour, but knockdown of both SP1 and miR‐326 dramatically boosted the growth of 143B tumour. E, The molecular mechanism by which SP1/miR‐326 regulated the proliferation and metastasis of OS. Sp1 epigenetically down‐regulated miR‐326 by recruiting HDAC1. Down‐regulated miR‐326 caused the overactivation of Hedgehog signalling pathway, resulting in the rapid proliferation, suppressed apoptosis and enhanced metastasis of OS. **P < .01, ***P < .001 vs control group

Transfection with SP1‐siRNA vector decreased SP1 but increased miR‐326 expression in 143B‐cell‐xenografted tumour, as indicated by the results form PCR assay (*P* < .001, Figure 7A). Treatment with miR‐326 inhibitor (antagonist) abolished the increased of miR‐326 expression caused by SP1 knockdown. SP1 knockdown inhibited 143B tumour growth in nude mice (*P* < .01, Figure 7B). However, the inhibition of tumour growth was abolished by the reduction of miR‐326. As indicated by immunohistochemistry, SMO and Ki67 protein abundances were decreased with SP1 knockdown. Knockdown of both SP1 and miR‐326 seemed to had no effect on SMO and Ki67 protein abundances (Figure 7C). This study investigated the metastasis of 143B cells to lung tissues using HE staining assay. 143B tumour belongs to solid tumour. It is easy to distinguish a solid tumour from lung tissues in which there are full of pulmonary alveoli. In lung tissues, SP1 knockdown was associated to smaller size of 143B tumour (Figure 7D), but knockdown of both SP1 and miR‐326 dramatically boosted the growth of 143B tumour. Figure 7E shows the molecular mechanism by which SP1/miR‐326 regulated the proliferation and metastasis of OS. The molecular mechanism was described and disused in following discuss section.

## 4 DISCUSSION

Transcription factor Sp1 has been extensively reported to increase expression of many oncogenes, thereby promoting the progression of OS and other types of tumour.^18^, ^19^ However, there are seldom studies investigating the transcriptional effect of Sp1 on miRNA. Mounting evidence has indicated that many miRNAs play important role in tumour occurrence and development.^20^, ^21^, ^22^ To understand the regulatory effect of Sp1 on miRNA, this study knocked down SP1 in MG63 and 143B cells and then performed miRNA microarray assay. Results showed that expression of multiple miRNAs was influenced by Sp1 in OS. Among them, miR‐326 has been implicated in the OS pathogenesis. Cao et al^21^ suggested that miR‐326 is a potential diagnostic and prognostic marker for OS, because miR‐326 expression was significantly decreased in OS, and patients with a lower expression of miR‐326 tended to have distant metastasis and a more advanced clinical stage. Wang et al^22^ found that small nucleolar RNA host gene 1, a long non‐coding RNA, increased expression of nin one binding protein (NOB1) in OS through sponging miR‐326. NOB1 is a oncogene, which contributes to cell growth, migration and invasion in OS. This study showed that miR‐326 was negatively regulated by Sp1, because Sp1 knockdown conversely increased miR‐326 expression. Since Sp1 was up‐regulated in OS compared to the normal bone tissues, up‐regulated Sp1 probably caused the deficiency of miR‐326 in OS.

Sp1 in most cases promotes gene expression; however, miR‐326 was negative regulated by Sp1. Previous study suggested that Sp1 not only functions as a transcription factor, but also participates in epigenetic modification by recruiting HDACs.^23^, ^24^ In renal cell carcinoma, loss of the Sp1‐HDAC1 complex is associated with elevated histone H3 acetylation at GM2‐synthase gene promoter, resulting in increased expression of GM2‐synthase and faster proliferation of tumour.^23^ In breast cancer, overactivation Sp1 caused by HER2 induced the accumulation of HDAC1 at MIR146A gene promoter, which decreased miR146A expression and consequently increased its oncogenic targets, interleukin‐1 receptor‐associated kinase 1 and the chemokine receptor CXCR4.^24^ This study found that activities of HDAC1, 2 and 3 were increased in OS tissues. In accordance with Sp1 knockdown, silencing HDAC1, but not HDAC 2 and 3, increased miR‐326 expression, suggesting that HDAC1 is involved in the regulation of miR‐326 expression. Results from ChIP assay further revealed that Sp1 recruited HDAC1 to miR‐326 promoter region, resulting in histone deacetylation. Histone deacetylation is in company with the inhibition of gene expression. Our data collectively suggested that Sp1‐HDAC1 complex down‐regulates miR‐326 expression by inducing the histone deacetylation at the promoter region.

A clinical analysis suggests that a lower expression of miR‐326 is correlated with a higher rate of distant metastasis and more advanced clinical stage of OS.^21^ OS metastasis is the leading cause of cancer‐related deaths. In this study, knockdown of Sp1 or overexpression of miR‐326 inhibited cell viability, proliferation, migration and invasion of OS cells. However, silencing miR‐326 reversed these inhibitory effects on OS resulted from Sp1 knockdown. Moreover, the metastasis of OS to lung tissues in nude mice was inhibited with Sp1 knockdown; however, the loss of miR‐326 strengthened the metastasis and growth of OS with Sp1 knockdown. These results suggested that miR‐326 plays important role in various cancer‐promoting effects of Sp1, especially Sp1‐induced metastasis of OS.

Accumulating evidence indicated that Hedgehog signalling pathway is a robust signal stimulating OS tumourigenesis and metastasis.^25^, ^26^ This study showed that Smoothened (Smo), a up‐stream activator of Hedgehog signalling pathway, is a target of miR‐326. Up‐regulation of miR‐326 reduced expression of Smo as well as its down‐stream target Gli1. Gli1 is a transcription factor that regulates expression of genes, including Ptch1, cyclin D1, Bcl‐2 and MMP9.^25^, ^26^ These genes are involved in the proliferation, invasion and metastasis of tumour. Sp1‐mediated down‐regulation of miR‐326 probably triggered overactivation of Hedgehog signalling pathway. Indeed, silencing Sp1 inhibited the activation of Hedgehog signalling pathway, while the depletion of miR‐326 via the inhibitor abolished the inhibitory effect.

In this study, we performed a rescue study by transfection with miR‐326 inhibitor to counteract the increase of miR‐326 cause by silencing Sp1. However, the suppressive effect of miR‐326 inhibitor was much stronger than the promoting effect of Sp1 knockdown on miR‐326. Thus co‐transfection with miR‐326 inhibitor and Sp1‐siRNA still resulted in the down‐regulation of miR‐326. In addition to SMO that has been identified in this study, many oncogenic proteins such as Sp1, ELK1 and NOB1 are confirmed as the targets or miR‐326.^27^, ^28^, ^29^ The reduction of miR‐326 probably increases the expression of these oncogenic proteins, resulting in enhanced proliferation, migration and invasion of OS cells.

In conclusion, this study revealed that Sp1 epigenetically down‐regulated miR‐326 by recruiting HDAC1. Down‐regulated miR‐326 caused the overactivation of Hedgehog signalling pathway, resulting in the rapid proliferation, suppressed apoptosis and enhanced metastasis of OS. Therefore, miR‐326 plays a key role in the cancer‐promoting effect of Sp1 in OS.

## CONFLICT OF INTEREST

None.

## AUTHOR CONTRIBUTIONS

**Jiang‐Hu Huang:** Data curation (equal); investigation (equal); writing‐review and editing (supporting). **Yang Xu:** Data curation (equal); methodology (equal); writing‐original draft (equal); writing‐review and editing (equal). **Fei‐Yue Lin:** Conceptualization (equal); data curation (equal); formal analysis (equal); funding acquisition (equal); investigation (equal); resources (equal); software (equal); supervision (equal); validation (equal); visualization (equal); writing‐original draft (equal); writing‐review and editing (equal).

## Data Availability

The data generated/analyzed in the present study are available upon reasonable request from the corresponding author.
